# Design and Effects of the Cinnamic Acids Chemical Structures as Organocatalyst on the Polymerization of Benzoxazines

**DOI:** 10.3390/polym12071527

**Published:** 2020-07-09

**Authors:** Rocío B. Rodríguez, Daniela Iguchi, Rosa Erra-Balsells, M. Laura Salum, Pablo Froimowicz

**Affiliations:** 1 Design and Chemistry of Macromolecules Group, Institute of Technology in Polymers and Nanotechnology (ITPN), UBA-CONICET, FADU, University of Buenos Aires, Intendente Güiraldes 2160, Pabellón III, subsuelo, Ciudad Universitaria, Buenos Aires C1428EGA, Argentina; rocio.rodriguez@fadu.uba.ar (R.B.R.); daniela.iguchi@fadu.uba.ar (D.I.); 2 Departamento de Química Orgánica, Facultad de Ciencias Exactas y Naturales, Universidad de Buenos Aires, Pabellón II, 3er piso, Ciudad Universitaria, Buenos Aires C1428EHA, Argentina; erra@qo.fcen.uba.ar; 3 Facultad de Ciencias Exactas y Naturales, Centro de Investigación en Hidratos de Carbono (CIHIDECAR), Universidad de Buenos Aires, CONICET, Pabellón II, 3er piso, Ciudad Universitaria, Buenos Aires C1428EHA, Argentina

**Keywords:** chemical design, benzoxazines, *Z*-cinnamic acid derivatives, catalysts, benzoxazine polymerizations

## Abstract

This study focuses on the catalytic effect of the two geometric isomers of a cinnamic acid derivative, *E* and *Z*-forms of 3-methoxycinnamic acid (3OMeCA), analyzing the influence of their chemical structures. *E* and *Z*-3OMeCA isomers show very good catalytic effect in the polymerization of benzoxazines, decreasing by 40 and 55 °C, respectively, the polymerization temperatures, for catalyst contents of up to 10% w/w. Isothermal polymerizations show that polymerizations are easily realized and analyzed at temperatures as low as 130 °C and at much shorter times using *Z*-3OMeCA instead of *E*-3OMeCA. Thus, both cinnamic acids are good catalysts, with *Z*-3OMeCA being better. The molecular reasons for this difference and mechanistic implications in benzoxazine polymerizations are also presented.

## 1. Introduction

**Scheme 1:**
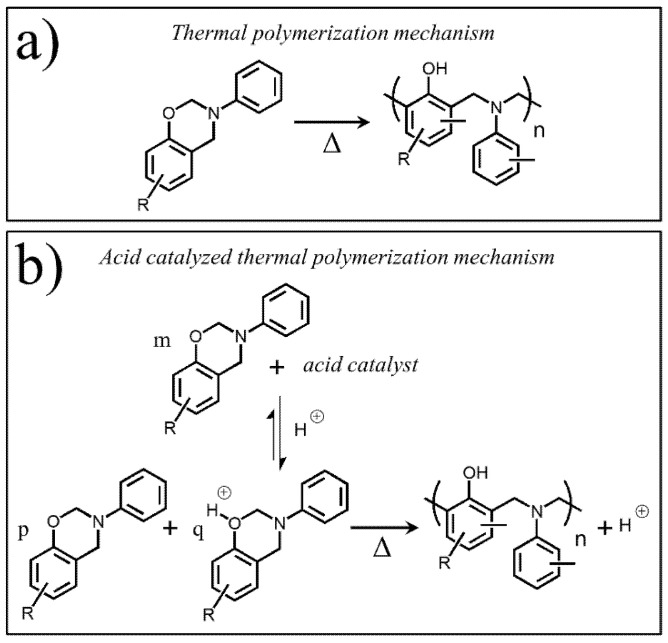
Accepted simplified benzoxazine polymerization mechanisms. (**a**) Thermal polymerization mechanism, only induced by heat; and, (**b**) classic acid catalyzed thermal polymerization mechanism, heat and acidic compounds participate to initiate the polymerization.

Benzoxazines and polybenzoxazines, relatively new classes of resins and polymers [1], are increasingly attracting scientific and industrial interests due to a set of unusual and yet very desirable properties, such as high chemical [2] and thermal resistance [3,4], low flammability [5,6], near-zero shrinkage throughout polymerization [7], and excellent adhesive [8], and mechanicals properties [9]. Despite these advantages, the application of polybenzoxazines remains, in some cases, relatively impractical given the required high polymerization temperatures (*T*_p_), which implies the need of special precautions and high energy consumption. Hence, decreasing their *T*_p_ is highly desired. Based on this need and the fantastic benzoxazine molecular design flexibility, clever design of sophisticated catalyst-containing benzoxazines have been reported [10,11,12]. In general, benzoxazines bearing these built-in catalysts are efficient at lowering the *T*_p_ as well as reducing or eliminating the need of homogeneously mixing external catalysts with the resins to be polymerized. However, they might turn out to be not as practical or economical as expected to be exploited at industrial level, due to the high synthetic complexity required for obtaining the resin, among other aspects. Conversely, and in a similar fashion as additives are added into the reaction systems before polymerization [13,14,15], the use of external catalysts still remains as a practical, viable, and valuable alternative for optimizing polymerization conditions. In this regard, it is well-known that benzoxazine polymerizations follow an intrinsic self-initiating thermal ring-opening polymerization mechanism [16], which can easily be acid catalyzed in presence of acidic compounds [16,17,18] as shown in Scheme 1.

The mentioned benzoxazine chemical flexibility has also allowed the utilization of phenolic derivatives obtained from renewable resources for synthetizing new monomers [19,20,21]. Pioneering works in that direction used cardanol [22], guaiacol [23], and cinnamic derivatives [24]. The latter ones are of particular interest due to the two geometric isomers in which they are found, *E*- and *Z*-forms [25]. Comí and coworkers reported the synthesis of mono-oxazine benzoxazines using the *E*-isomer of substituted and unsubstituted cinnamic acid derivatives [24]. It was found that, whereas cinnamic-containing monomers bear low *T*_p_, they are very unstable under polymerization conditions, making impossible to obtain the corresponding thermosets. Thus, based on this precedent and with the extra interest in overcoming these difficulties to establish a practical and economical approach, we propose in this work the use of cinnamic acid derivatives as catalysts for benzoxazine polymerization instead of incorporating them as a constitutive moiety of the resins. Importantly, this work presents a comparative study on the catalytic performance between the two geometric isomers, *E*- and *Z*-form, of the cinnamic catalysts.

## 2. Experimental

### 2.1. Materials

3-pentadecylphenol (technical grade, 90%), aniline (99%), paraformaldehyde (powder, 95%), *E-*3-methoxycinnamic acid (predominantly *E*, 99%) and *n-*butylamine (99.5%) were purchased from Sigma-Aldrich (St. Louis, MO, USA) and used as received. All organic solvents were HPLC grade and purchased from Sigma-Aldrich (St. Louis, MO, USA).

### 2.2. Synthesis of Z-3-Methoxycinnamic Acid (Z-3OMeCA)

*Z-*3-methoxycinnamic acid (*Z-*3OMeCA) was synthetized and isolated following a reported procedure [26]. Briefly, a stoichiometric mixture of *E-*3-methoxycinnamic acid and *n-*butylamine was irradiated overnight in solution (3.3 × 10^−3^ M, acetonitrile:methanol, 98:2). The obtained product was then isolated and purified as described in the reported works. Reaction yield: 60%.

*Z*-3OMeCA ^1^H NMR (500 MHz, DMSO-*d*_6_) δ: 12.55 (s, 1H, –COOH), 7.27 (m, 2H, Ar), 7.13 (d, 1H, Ar), 6.92 (dd, 1H, Ar), 6.86 (d, J = 12.8 Hz, 1H, H_a_), 5.96 (d, J = 12.8 Hz, 1H, H_b_), 3.75 (s, 3H, –OCH_3_). ^13^C NMR (126 MHz, DMSO-*d*_6_) δ: 167.58, 158.86, 139.77, 136.16, 129.12, 122.03, 121.37, 114.73, 114.49, 55.03. FT-IR (ATR) ν (cm^−1^): 1686 (C=O stretching), 1625 (C=C stretching), 1227 (C–O stretching). m.p.: 39–41 °C.

### 2.3. Characterization of E-3-Methoxycinnamic Acid (E-3OMeCA)

For comparative reasons, *E*-3OMeCA was also characterized. *E*-3OMeCA ^1^H NMR (500 MHz, DMSO-*d*_6_) δ: 12.41 (s, 1H, -COOH) 7.57 (d, J = 16.0 Hz, 1H, H_a_), 7.33 (t, 1H, Ar), 7.26 (m, 2H, Ar), 6.99 (dd, 1H, Ar), 6.56 (d, J = 16.0 Hz, 1H, H_b_), 3.80 (s, 3H, –OCH_3_). ^13^C NMR (126 MHz, DMSO-*d*_6_) δ: 167.57, 159.60, 143.90, 135.66, 129.93, 120.78, 119.55, 116.27, 112.90, 55.22. FT-IR (ATR) ν (cm^−1^): 1674 (C=O stretching), 1628 (C=C stretching), 1214 (C–O stretching). m.p.: 117–199 °C.

### 2.4. Synthesis of Benzoxazine Monomer (PDP-a)

PDP-a benzoxazine monomer was synthetized following a modified procedure from reported literature [27]. Briefly, in a round-bottom flask equipped with a magnetic stirrer and a condenser a mixture of 3-pentadecylphenol (3.05 g, 0.01 mol) and aniline (0.93 g, 0.01 mol) was heated at 70 °C until a homogenous system was obtained. Then, paraformaldehyde (0.60 g, 0.02 mol) was added and the mixture was heated at 100 °C for 3 h. After cooling, chloroform in excess was added to reaction mixture and then washed 3 times with 1 M NaOH aqueous solution and finally with distilled water. The product was dried over anhydrous Na_2_SO_4_, filtered, and then chloroform was removed by evaporation. Reaction yield: 65%.

PDP-a ^1^H NMR (600 MHz, Chloroform-*d*) δ: 7.27 (t, 2H, Ar), 7.11 (d, 2H, Ar), 6.93 (m, 2H, Ar), 6.72 (dd, 1H, Ar), 6.64 (d, 1H, Ar), 5.35 (s, 2H, O–C*H*_2_–N), 4.61 (s, 2H, Ar–C*H*_2_–N), 2.51 (t, 2H, Ar–C*H*_2_–aliphatic chain), 1.68–1.42 (m, 2H, Ar-CH_2_-C*H*_2_-aliphatic chain), 1.39–1.12 (m, 24H, aliphatic C*H*_2_ protons), 0.89 (t, 3H, terminal -C*H*_3_). ^13^C NMR (151 MHz, Chloroform-*d*) δ: 154.37, 148.69, 143.22, 129.40, 126.59, 121.45, 121.25, 118.35, 118.16, 116.80, 79.56 (O–*C*H_2_–N), 50.43 (Ar–*C*H_2_–N), 35.82, 32.08, 31.44, 29.85, 29.82, 29.73, 29.66, 29.51, 29.47, 22.84, 14.24. FT-IR (KBr disc) ν (cm^−1^): 2965, 2916, 2848 (aliphatic chain C–H stretching), 1227 (C–O–C antisymmetric stretching), 1498 (trisubstituted aromatic ring), 990 and 968 (trisubstituted aromatic ring and benzoxazine related band). m.p.: 67–69 °C.

### 2.5. Preparation of Cinnamic Catalyst/Benzoxazine Resin Mixtures

PDP-a benzoxazine resin was mixed with different ratios of *E*-3OMeCA or *Z*-3OMeCA by gently grinding both solids in a mortar with pestle, forming PDP-a/*E*-3OMeCA or PDP-a/*Z*-3OMeCA mixtures, respectively. The specific catalyst contents in each mixture were of 0; 2.5; 5.0; and, 10.0% w/w. All mixtures were subsequently subjected to the polymerization studies opportunely described.

### 2.6. Instrumentation

*Photoisomerization*. Irradiations were at ~300 nm in a photoreactor using 3 lamps Rayonet RPR 3000 Å lamp.

*Spectroscopic characterization*. *Z/E-3OMeCA:*^1^H and ^13^C NMR spectra were recorded using a Bruker AVANCE II 500 NMR spectrometer operating at 500.14 and 125.76 MHz, respectively. DMSO-*d*_6_ was used as solvent. Chemical shift values are reported in ppm and the coupling constants are given in Hz. Fourier transform infrared (FT-IR) spectra were recorded using a Nicolet IS50 spectrophotometer, ATR mode, accumulating and averaging 16 scans at a resolution of 4 cm^−1^. *PDP-a*: ^1^H and ^13^C NMR spectra were recorded using a Bruker AVANCE III 600 operating at 600 and 150.8652 MHz, respectively. Chloroform-*d* was used as solvent. FT-IR spectra were recorded using a IR Affinity-1 Shimadzu spectrophotometer with KBr pellets, absorbance mode, accumulating and averaging 40 scans at a resolution of 4 cm^−1^.

*Thermal analysis*. Differential scanning calorimeter (DSC) were carried out using a Shimadu DSC-60 under a nitrogen flow rate of 30 mL/min, sample mass of 1.5 ± 0.2 mg, with aluminum pans. Nonisothermal DSC studies were always at the heating rate of 10 °C/min. In the cases of activation energy of polymerizations, the following heating rates of 2.5; 5; 10; 15; and 20 °C/min were applied. Isothermal studies were at 130, 150, 170 and 190 °C, these temperatures were reached at the heating rate of 10 °C/min. To make results comparable, time zero was always set as the same temperature when starting the experiment.

*Titration*. Potentiometric titrations were carried out using a Arcano PH-013M pHmeter (calibrated with standard buffers) with automatic temperature compensation.

## 3. Results and Discussions

**Figure 1:**
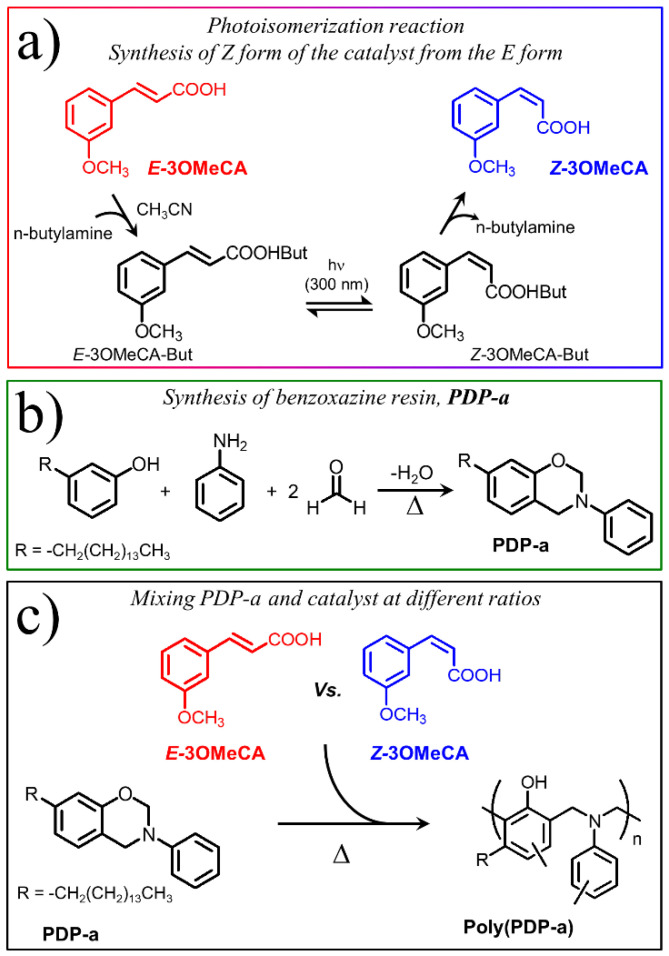
(**a**) General approach for synthesizing *Z*-forms of cinnamic derivatives. (**b**) Synthesis of benzoxazine monomer (PDP-a). (**c**) PDP-a polymerizations catalyzed by the cinnamic catalysts.

Figure 1 shows the chemical structures of the compounds used in this work and how they were synthesized and utilized. The isomer pair studied as catalysts is the *E*- and *Z*-3-methoxycinnamic acid (*E*-3OMeCA and *Z*-3OMeCA), where the *Z*-form was synthesized photoisomerizing [26] the inexpensive and commercially available *E*-3OMeCA (Figure 1a). The simple mono-oxazine benzoxazine synthetized by Mannich condensation using aniline, 3-pentadecylphenol, and paraformaldehyde [27] (hereinafter named PDP-a, Figure 1b) is used as the polymerizable resin. The comparative catalytic study was realized mixing each catalyst with the resin in solid state, at different ratios, and then subjected to several polymerization conditions (Figure 1c).

**Figure 2:**
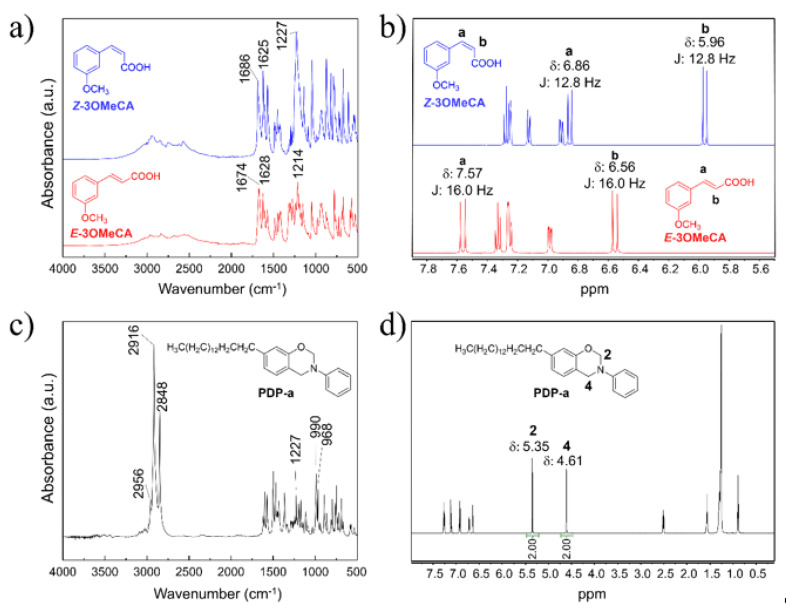
(**a**) FT-IR spectra of *E*- and *Z*-3OMeCA. (**b**) ^1^H NMR spectra of *E*- and *Z*-3OMeCA. (**c**) FT-IR spectrum of PDP-a. (**d**) ^1^H NMR spectrum of PDP-a.

The isolated final products of each synthesis were characterized by FT-IR and ^1^H NMR. All spectral information is shown in Figure 2. For comparative reasons, *E*-3OMeCA is also presented in the characterization.

FT-IR spectra of *Z*-3OMeCA and *E-*3OMeCA presented the typical absorptions bands assigned to their own functional groups, which are reported to differ significantly between the *E* and *Z* isomers [28]. As seen in Figure 2a, the top spectrum shows absorbance bands at 1686, 1227, and 1625 cm^−1^ assigned to C=O, C–O, and C=C corresponding to *Z*-3OMeCA, while the bottom spectrum shows 1674, 1214, and 1628 cm^−1^ assigned to C=O, C–O, and C=C corresponding to *E-*3OMeCA. Similarly, ^1^H NMR spectra of *Z*-3OMeCA and *E-*3OMeCA presented their typical signals, where the most noticeable ones are the two corresponding to the vinylic protons, marked in Figure 2b as H_a_ and H_b_. As can be seen in the figure, the top spectrum shows two doublets at 6.86 and 5.96 ppm with a coupling constant of 12.8 Hz assigned to H_a_ and H_b_ of *Z*-3OMeCA, while the bottom spectrum shows two doublets at 7.57 and 6.56 ppm with a coupling constant of 16.0 Hz assigned to H_a_ and H_b_ of *E-*3OMeCA [26,29].

The FT-IR spectrum of PDP-a is shown in Figure 2c, where the most important absorption bands to identify the compound are seen at 968, 990, and 1227 cm^−1^ and assigned to the typical benzoxazine related band, trisubstituted aromatic ring, and C–O–C antisymmetric stretching, while those at 2956, 2916, and 2848 cm^−1^ are attributed to the different C-H stretching of the aliphatic side chain. Similarly, ^1^H NMR spectrum of PDP-a indicates the successful synthesis of the resin, see Figure 2d. The two singlets observed at 5.35 and 4.61 ppm in the spectrum are assigned to the two –CH_2_– forming the oxazine ring, referred to as H_2_ and H_4_. The integration ratio of these two signals are equal and equivalent to two protons per signal, thus revealing perfect closure of the oxazine ring.

**Figure 3:**
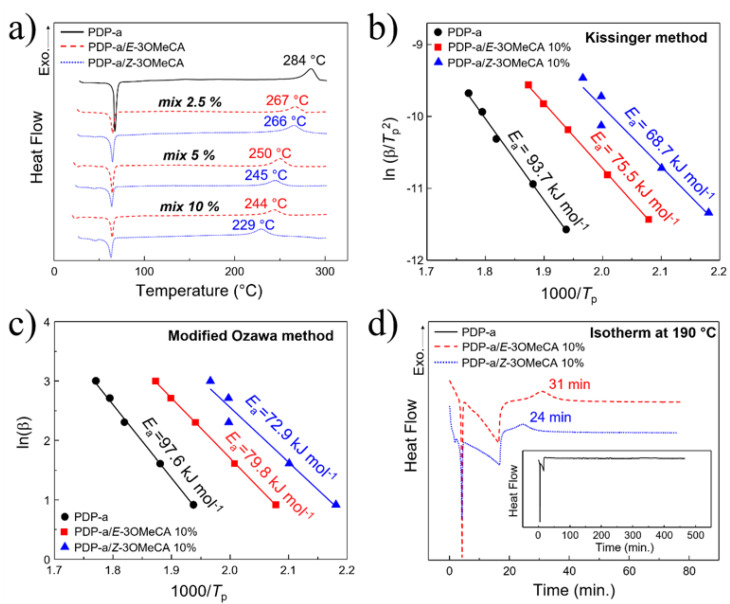
(**a**) DSC thermograms of PDP-a and PDP-a/catalyst mixtures at different ratios. Plots generated following the Kissinger (**b**) and modified Ozawa (**c**) methods for calculating the activation energy of catalyzed and uncatalyzed polymerization reactions. (**d**) Isothermal DSC thermograms at 190 °C for catalyzed and uncatalyzed (inset) polymerizations of PDP-a.

The catalytic activity of each cinnamic acid derivative was evaluated by DSC (Figure 3). Figure 3a shows that the *T*_p_ decreases when the amount of catalyst in the mixtures prepared beforehand increases. Similar behavior is observed for both catalysts. Notably, at higher catalyst ratios, higher differences between the *T*_p_ of the two catalyzed systems are observed. In all cases, the *Z*-form catalyst exhibited the lowest *T*_p_. Thus, Z-3OMeCA is a more effective catalyst than *E*-3OMeCA.

The stronger catalytic activity of *Z*-3OMeCA is further inferred from Figure 3b,c, where activation energies of uncatalyzed and catalyzed (catalyst at 10% w/w) polymerization reactions, calculated by the Kissinger and modified Ozawa methods, present the increasing order *Z*-3OMeCA-catalyzed system < *E*-3OMeCA-catalyzed system < uncatalyzed system. It is worth mentioning that the activation energy values obtained in this work are much lower than those obtained for some other benzoxazine resins reported in the literature, such as 107 (Kissinger) and 109 kJ/mol (Ozawa) for a built-in catalyst containing naphthoxazine [10], 169 kJ/mol (Kissinger) and 167 kJ/mol (Ozawa) for a symmetric amide-functional benzoxazine [30], 121 kJ/mol (Kissinger) and 122 kJ/mol (Ozawa) for an asymmetric amide-imide functional benzoxazine [30], and 101 kJ/mol (Kissinger) and 116 kJ/mol (Ozawa) for a conventional mono-oxazine benzoxazine without any catalytic system [31]. Based on these results, we also developed an interest in understanding how these systems would behave under isothermal polymerization conditions. As expected, the *Z*-3OMeCA-catalyzed system presented a higher polymerization rate than the other systems for polymerization performed at 190 °C (Figure 3d). It must be mentioned that no exotherm is detected for the uncatalyzed system (neat PDP-a) at this temperature, although complete polymerization was demonstrated to happen after 450 min as investigated in a second DSC run performed at 10 °C/min.

**Figure 4:**
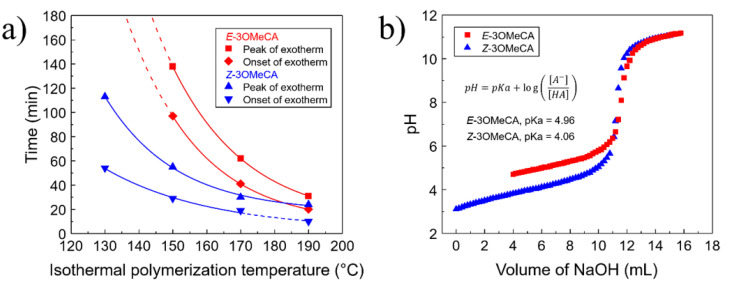
(**a**) Plot of time as a function of isothermal polymerization temperatures of peaks and onsets for the *E*-3OMeCA (■, ◆, respectively) and *Z*-3OMeCA (▲, ▼, respectively) catalyzed polymerizations of PDP-a, catalyst concentration: 10% w/w. (**b**) Titration curves for each catalyst, *E*-3OMeCA (■) and *Z*-3OMeCA (▲). Solvent: H_2_O:MeOH 90:10.

From a processing standpoint or with industrial interests, it is worth mentioning that a wide processing window is observed between melting and polymerization of the resin in all catalyzed and uncatalyzed non-isothermal studies (Figure 3a). Complementary isothermal polymerizations at different temperatures revealed that, as expected, higher polymerization temperatures make the exotherm maxima and onsets occur faster. Results are presented in Figure 4a for each catalyzed system. The behavior of uncatalyzed polymerizations is not presented because exotherms were not detectable at these temperatures.

Figure 4a shows that, for both catalysts, polymerization exotherm maximum and onset times as a function of isothermal treatment temperatures follow exponential decays. Notably, *Z*-3OMeCA-catalyzed polymerization initiates instantly for the isotherm at 190 °C as the onset occurs within the end of the heating ramp (see Figure 3d). Clearly, the two polymerization parameters are easily adjustable modifying the temperatures of the isothermal polymerizations, as demonstrated in Figure 4a. The stronger catalytic activity of *Z*-3OMeCA compared to *E*-3OMeCA is again evidenced since exotherm maxima and onsets occur faster under identical isothermic conditions. Note that no exotherm was detected for the *E*-3OMeCA-catalyzed polymerization throughout the isothermic treatment at 130 °C.

Chemically, a question must be answered at this stage, why is *Z*-3OMeCA a more effective catalyst than *E*-3OMeCA for benzoxazine polymerization? It has been reported that organic acids catalyze benzoxazine polymerization [17]. Most studies present the influence of acid/acidic catalysts as a function of concentration [11,16,17,32,33,34,35]. In this regard, Figure 3a presents a similar study for the two cinnamic catalysts investigated in this work, where higher acid concentrations induce greater lowering of *T*_p_. Nevertheless, in this study, we also demonstrate that the acid with the highest acidity (smaller pKa values) presents the strongest catalytic effect. Figure 4b shows the titration curves from which pKa values of 4.06 and 4.96 were calculated for *Z*-3OMeCA and *E*-3OMeCA, respectively. Because of the catalysts’ solubility (Table 1), titrations were carried out using a H_2_O:MeOH 90:10 mixture as solvent. While *Z*-3OMeCA was soluble in this mixture, *E*-3OMeCA formed a homogeneous solution only after adding 4 mL of NaOH titration solution. Table 1 also shows that *Z*-3OMeCA is more soluble than *E*-3OMeCA in CHCl_3_, suggesting a better compatibility with the hydrophobic resin, which is highly soluble in this solvent. This result reinforces the fact that *Z*-3OMeCA might be seen as a better catalyst than *E*-3OMeCA for polymerizing PDP-a.

**Scheme 2:**
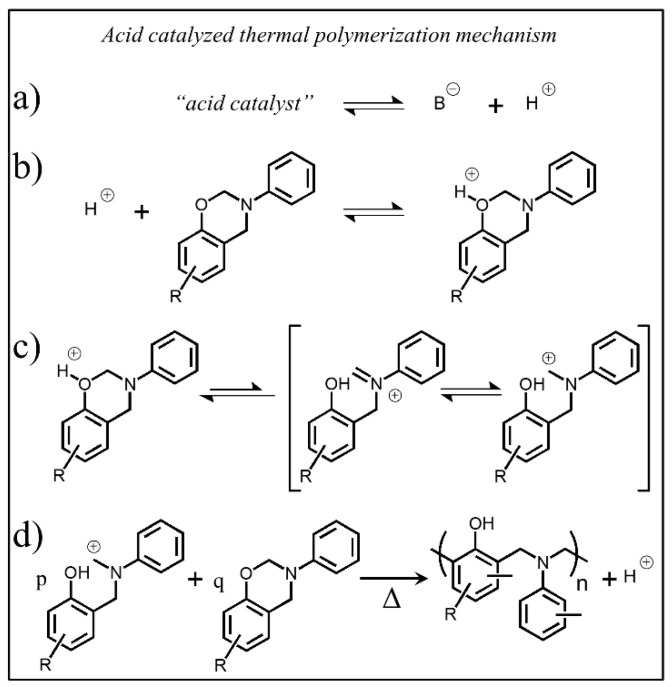
Simplified step-by-step acid catalyzed benzoxazine polymerization mechanism. (**a**) Dissociation equilibrium of the acid catalyst. (**b**) Protonation of the benzoxazine species. (**c**) Ring-chain tautomeric equilibrium of benzoxazine resins. (**d**) Acid catalyzed benzoxazine polymerization upon thermal treatment.

Dependency upon acid strength is easily interpreted considering that both catalysts are cinnamic acids, which are weak acids presenting their own dissociation equilibria. Then, when both catalysts are under the same reaction conditions, at a given pH, they will dissociate differently. Clearly, the stronger acid will present a greater ratio of its dissociated products, conjugated base and proton, than the weaker one. In other words, under the same condition, *Z*-3OMeCA will generate a higher ratio of protons in the reaction system than *E*-3OMeCA. This higher ratio of protons in the reaction system might be considered as a virtual manner of increasing the concentration of the catalyst. This rationalization is acceptable because, as shown in Scheme 2, the actual catalyst is the proton. Greatening the proton concentration, by whichever method, shifts the equilibrium producing a higher amount of the protonated benzoxazine species (Scheme 2b), which in turn generates the more reactive cationic species, thus increasing the reaction rate of polymerization upon thermal treatment (Scheme 2d). The mechanism proposed herein is in full agreement with those previously accepted accelerated (or activated) polymerization mechanisms catalyzed by acid and acidic compounds intentionally added [16] or present as impurities [1] reported hitherto.

**Figure 5:**
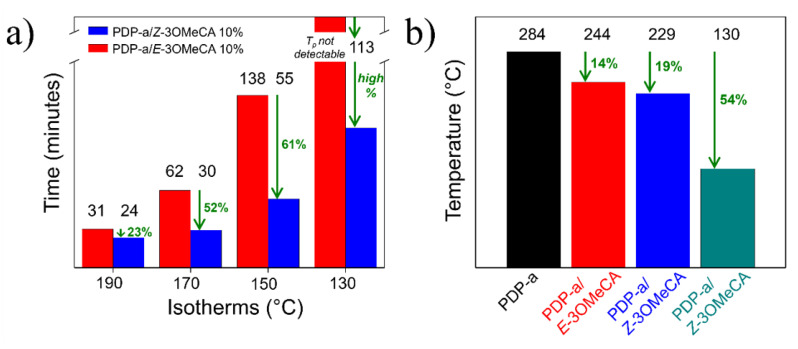
Percentage enhancement (green arrows and values) when comparing: (**a**) time to reach exotherm maxima under each isothermal treatment for systems catalyzed by *E*-3OMeCA (red bars) and *Z*-3OMeCA (blue bars); and, (**b**) temperature reduction for uncatalyzed (black bar) and *E*-3OMeCA (red bar) and *Z*-3OMeCA (blue bar) catalyzed systems under non-isothermal conditions, and when isothermally catalyzed by *Z*-3OMeCA (teal bar).

A very impressive result is observed in Figure 5, which shows that polymerization times and temperatures are easily adjustable using the catalysts. Figure 5a presents a quantitative comparison of polymerization times between the *Z*-3OMeCA and *E*-3OMeCA-catalyzed systems under isothermal treatments. While *E*-3OMeCA-catalyzed polymerizations under isothermic conditions at 190, 170, and 150 °C take 31, 62, and 138 min, and are no longer detectable at 130 °C, it takes 24, 30, 55, and 113 min for *Z*-3OMeCA-catalyzed systems. Thus, polymerizations catalyzed by *Z*-3OMeCA are 23%, 52%, and 61% faster than when catalyzed by *E*-3OMeCA. At polymerization temperatures as low as 130 °C, *Z*-3OMeCA presents the extra advantage of being the only catalyst producing a detectable polymerization exotherm.

Figure 5b shows that a marked reduction of the *T*_p_ is also achievable using cinnamic-based catalysts. Enhancements of 14% and 19% are observed in the figure for catalysts *E*-3OMeCA and *Z*-3OMeCA under nonisothermal conditions. Moreover, comparing those systems with that under isothermal conditions at 130 °C, which is only possible for *Z*-3OMeCA catalyst, a remarkable reduction of up to 54% is perceived.

## 4. Conclusions

In summary, two geometric isomers of cinnamic acid derivatives, the *E* and *Z* forms of 3-methoxycinnamic acid (3OMeCA), were studied as catalysts for benzoxazine polymerizations. It was found that both isomers are good catalysts for polymerization reactions. Both catalysts efficiently decrease the *T*_p_, lowering it by about 15% and 20% for catalyst contents of up to 10% w/w and for *E*-3OMeCA and *Z*-3OMeCA, respectively. More importantly, isothermal polymerizations showed that *Z*-3OMeCA-catalyzed polymerizations were in all cases much faster than those catalyzed by *E*-3OMeCA. Therefore, *Z*-3OMeCA exhibited a higher catalytic activity than *E*-3OMeCA. The observed polymerization times made it possible to easily perform and analyze polymerizations at temperatures as low as 130 °C in under 2 h. It must be mentioned that processing times can easily be controlled by varying different variables or a combination of variables, such as the type of catalyst, amount of catalyst, or polymerization temperature. This cost-efficiency, practicality, and easy versatility achievable using these catalysts might make this technology highly desirable from an industrial standpoint.

## Figures and Tables

**Table 1:** Qualitative solubility tests for *E*-3OMeCA and *Z*-3OMeCA ^a^.

\	CHCl_3_	MeOH	DMSO	H_2_O
***E*-3OMeCA**	−/±	✓	✓	✘
***Z*-3OMeCA**	✓	✓	✓	−/±

^a^: ✓ Good solubility. ± Low solubility. −/± Very low solubility. ✘ Insoluble.
